# Place de l’histopathologie dans le diagnostic et la prise en charge des méningiomes intracrâniens. Expérience hospitalière à Kinshasa (République démocratique du Congo)

**DOI:** 10.48327/mtsi.v5i4.2025.647

**Published:** 2025-10-22

**Authors:** Elie NKAMBA KAPIAMBA, Olive KISILE MIKUO, Glennie NTSAMBI EBA, Jean-Marie KABONGO MPOLESHA, Bienvenu LEBWAZE MASSAMBA, Raphaël CHIRIMWAMI BULAKALI

**Affiliations:** 1 Département d’anatomie-pathologique de Kinshasa, Faculté de médecine, Université de Kinshasa, BP 123, République démocratique du Congo; 2 Département de chirurgie, Service de neurochirurgie de Kinshasa, Faculté de médecine, Université de Kinshasa, BP 123, République démocratique du Congo

**Keywords:** Méningiome, Tumeurs cérébrales, Imagerie cérébrale, Histopathologie, Kinshasa, République démocratique du Congo, Afrique subsaharienne, Meningioma, Brain tumors, Brain imaging, histopathology, Kinshasa, Democratic Republic of Congo, Sub-Saharan Africa

## Abstract

**Introduction:**

Les méningiomes sont les tumeurs cérébrales intracrâniennes les plus fréquentes, dont la nature histologique est généralement bénigne. Bien que l’histopathologie reste lexamen de référence dans le diagnostic et la classification des méningiomes, leur traitement dans les pays en voie de développement est fortement dépendant de la tomodensitométrie (TDM) et de l’imagerie par résonnance magnétique (IRM). Il est donc important d’avoir des résultats d’imagerie qui se rapprochent au mieux de la réalité. Nous avons mené cette étude dans l’objectif de déterminer l’apport de l’histopathologie dans le diagnostic des méningiomes intracrâniens dans les établissements de soins de santé tertiaires (ESS) de la ville de Kinshasa en République démocratique du Congo.

**Matériels et méthode.:**

Nous avons réalisé une étude transversale à visée descriptive dans les ESS disposant des services d’anatomie pathologie et de neurochirurgie dans la ville province de Kinshasa au cours d’une période de 10 ans. Nous avons exploité 102 dossiers de patients ayant été admis et opérés pour une tumeur cérébrale.

**Résultats:**

Le déficit neurologique et céphalique et l’atteinte du nerf optique étaient les principales plaintes chez respectivement 49 (48%) et 23 (22,5%) patients. Les hypothèses diagnostiques les plus évoquées étaient un méningiome chez 44 patients (43,1%), une tumeur cérébrale chez 28 (27,5%) et un processus intracrânien expansif chez 14 (13,7%). Quatre-vingt-quatorze patients (92,2%) ont réalisé une TDM et 7 (6,9%) une IRM. Les diagnostics les plus fréquents étaient un méningiome chez 40 patients (39,2%), une tumeur cérébrale chez 34 (33,3%) et un gliome chez 15 (14,7%). Des 36 patients (35%) ayant réalisé un examen histopathologique, 20 (56%) étaient des méningiomes, 5 (14%) des oligodendrogliomes. Sur les 20 cas de méningiomes diagnostiqués par l’histopathologie, 7 (35%) étaient des méningiomes fibroblastiques et 4 (20%) des méningiomes mixtes. L’imagerie médicale n’a permis le diagnostic de méningiome que dans 5 cas (25%). La majorité des méningiomes était de grade 1 (85%), suivi du grade 3 (10%).

**Conclusion:**

Malgré l’importance de l’histopathologie dans l’évaluation et la connaissance du grade des méningiomes, la majorité de nos patients n’y a pas recours. Il y a un écart important entre les conclusions de l’imagerie et celles de l’histopathologie des méningiomes, ce qui nécessite une sensibilisation des différents prestataires.

## Abstract (en)

**Introduction:**

Meningiomas are the most common type of intracranial brain tumor and are usually benign. While histopathology is still considered the gold standard for diagnosing and classifying meningiomas, treatment in developing countries heavily relies on computed tomography (CT) and magnetic resonance imaging (MRI). Therefore, it is important to have imaging results that are as accurate as possible. The aim of this study was to determine the contribution of histopathology to the diagnosis of intracranial meningiomas in tertiary healthcare facilities (THFs) in Kinshasa, Democratic Republic of Congo.

**Materials and methods:**

We conducted a descriptive, cross-sectional study in THFs with pathology and neurosurgery departments in the city-province of Kinshasa, over a period of ten years. We analyzed 102 patient files of individuals admitted and operated on for a brain tumor.

Results. The main complaints were neurological and cephalic deficits and optic nerve damage in 49 (48%) and 23 (22.5%) patients, respectively. The most common diagnostic hypotheses were meningioma (43.1%), brain tumor (27.5%), and expansive intracranial process (13.7%). Eighty-four patients (92.2%) underwent CT scans, and seven (6.9%) underwent MRIs. The most common diagnoses were meningioma in 40 patients (39.2%), brain tumor in 34 patients (33.3%), and glioma in 15 patients (14.7%).

Of the 36 patients who underwent histopathological examination, 20 (56%) had meningiomas, and five (14%) had oligodendrogliomas. Of the 20 cases of meningioma diagnosed by histopathology, seven (35%) were fibroblastic meningiomas, and four (20%) were mixed meningiomas. Medical imaging allowed for a meningioma diagnosis in only five cases (25%). Most meningiomas were grade 1 (85%), followed by grade 3 (10%). Conclusion. Despite the importance of histopathology in evaluating and understanding meningioma grade, most of our patients do not undergo this procedure. There is a significant discrepancy between imaging findings and histopathological findings of meningiomas, which requires awareness among various healthcare providers.

## Introduction

Les méningiomes dérivent des cellules méningothéliales de la calotte arachnoïdienne et sont les tumeurs cérébrales primitives non gliales les plus répandues. Ils sont considérés comme les tumeurs intracrâniennes bénignes les plus courantes [6]. On note une augmentation de l’incidence globale aux États-Unis, passant de 4,52 à 8,3 pour 100 000 personnes entre 1998-2002 et 2010-2014 [11]. Cette augmentation s’expliquerait par la mise en place des nouvelles techniques de diagnostic. On assiste à une augmentation prédominante chez les personnes de plus de 70 ans chez lesquelles elle est 3,5 fois plus élevée. La littérature rapporte une incidence des méningiomes plus importante chez la femme avec une variabilité de 2 à 7/100 000 pour ces dernières et de 1 à 5/100 000 pour les hommes [16].

Plusieurs traitements ont été mis en place ces dernières années contre les méningiomes, dont la thérapie génique, la chimiothérapie moléculaire, les traitements hormonaux et l’irradiation multifaisceaux. Cependant, la chirurgie reste l’arme majeure du traitement des méningiomes dans les pays en voie de développement où elle est fortement dépendante de l’imagerie par tomodensitométrie (TDM) et par résonnance magnétique (IRM), d’où l’importance d’avoir des résultats d’imagerie qui se rapprochent de la réalité [4,8]. L’IRM joue un rôle crucial dans la détection des lésions, l’évaluation des complications liées à la tumeur et l’établissement de diagnostics différentiels des méningiomes intracrâniens. En outre, l’imagerie d’un méningiome permet un diagnostic précis et fiable dans la majorité des cas [14]. Plusieurs études ont montré une relation entre les caractéristiques de l’IRM et le grade des méningiomes, comme la présence d’une nécrose, d’une hémorragie tumorale, ou d’une interface tumeur-cerveau, le rehaussement hétérogène de la tumeur et l’œdème cérébral péri-tumoral [14]. Le pronostic du méningiome dépend de sa localisation, du grade histologique au moment de son diagnostic, de l’étendue de résection et de son agressivité biologique [5]. L’histopathologie reste un examen de référence pour le diagnostic et la classification des méningiomes. Elle joue un grand rôle dans la prise en charge et le suivi permettant la détermination du grade et du soustype histologique [9].

Les praticiens sont confrontés à des cas présentant des discordances entre les données cliniques, l’imagerie et l’histopathologie. Ainsi, un méningiome de grade élevé peut être découvert fortuitement par l’IRM chez un patient asymptomatique ou présentant des signes cliniques atypiques avec des symptômes inhabituels tels que des troubles cognitifs, alors que l’imagerie suggère un méningiome de grade modeste. Cette discordance entre imagerie et histologie est possible notamment dans certains sous-types histologiques tels que le méningiome sécrétoire qui peut avoir des aspects radiologiques trompeurs. Des artefacts sont possibles dans la technique histologique, notamment lors de l’exérèse chirurgicale ou de la préparation des lames, qui peuvent rendre l’interprétation histologique difficile. Mais il peut y avoir aussi des discordances liées à l’hétérogénéité intratumorale. Enfin, une discordance entre clinique et histologie est possible en fonction de variables comme l’âge du patient, ses antécédents médicaux ou la présence de comorbidité qui peuvent influencer la présentation clinique et l’évolution de la tumeur.

Nous avons mené cette étude afin de déterminer l’apport de l’histopathologie dans le diagnostic et la classification des méningiomes intracrâniens dans les hôpitaux de référence de la ville de Kinshasa, République démocratique du Congo (RDC), en comparaison avec les différentes approches diagnostiques cliniques et de l’imagerie médicale.

## Matériel et méthode

Nous avons réalisé une étude transversale descriptive, sur une période allant de janvier 2012 à décembre 2022, dans les établissements de soins de santé du niveau tertiaire (ESS), c’est-à-dire offrant un paquet des soins spécialisés et assurant la formation des médecins spécialisés. Ont été retenus les ESS qui disposaient des services d’anatomie pathologie et de neurochirurgie dans la ville province de Kinshasa (RDC).

Notre échantillon était constitué des patients admis dans le service de neurochirurgie pour une tumeur cérébrale et ayant réalisé un examen d’imagerie médicale (TMD, IRM et radiologie). Au total, 102 dossiers de patients ont été sélectionnés. Les critères d’inclusion étaient la disponibilité de la fiche du patient au service, la présence de toutes les données des variables d’intérêt dans cette fiche ou dans le registre des malades du service. Étaient retenus les patients ayant été opérés. Les données présentes dans le dossier des patients ont été rapportées dans une fiche électronique (KoboCollect) qui comprenait quatre sections:

- caractéristiques sociodémographiques (âge et sexe);
- caractéristiques cliniques (symptômes et hypothèses diagnostiques);
- caractéristiques paracliniques (examen d’imagerie réalisé et résultats);
- caractéristiques histopathologiques (résultat histopathologique et sous-types histologiques).

Les critères de diagnostic iconographiques ont concerné l’imagerie médicale. Les patients ont été soumis à des examens d’imagerie, incluant la TDM pour détecter les masses intracrâniennes, l’IRM pour fournir des images détaillées des tissus mous et différencier les méningiomes des autres types de tumeurs, et la radiographie pour évaluer l’extension osseuse des tumeurs.

Pour les critères de diagnostic anatomopathologiques, les échantillons de tissu tumoral ont été prélevés par exérèse chirurgicale et soumis à des analyses histopathologiques au laboratoire pour confirmer le diagnostic de méningiome, déterminer le type histologique, établir le grade du méningiome, ou poser un autre diagnostic. Il ne nous a pas été possible de répertorier les types de chirurgies réalisées selon la classification de Simpson en raison de l’absence de ces données dans la majorité des dossiers.

L’analyse histopathologique a été réalisée avec la coloration hématoxyline et éosine.

Les données ont été analysées en utilisant le logiciels SPSS® 27, après extraction sur Excel® 2016. Les variables qualitatives ont été exprimées sous forme de fréquences et de proportions et les variables quantitatives sous forme de moyenne et d’écart type.

## Résultats

**Figure 1:**
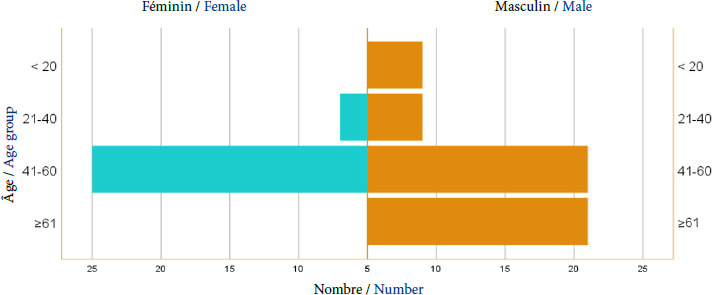
Répartition des patients porteurs de méningiome selon les tranches d’âge et le sexe

Sur les 102 patients de la série, l’âge moyen est de 47 (14-79) ans avec la proportion la plus élevée parmi les 41-60 ans (46 patients, soit 45,1%), suivi des plus de 61 ans (35%). Le sexe masculin était plus représenté, avec un effectif de 60 hommes (58,8%) (Fig. 1).

**Figure 2:**
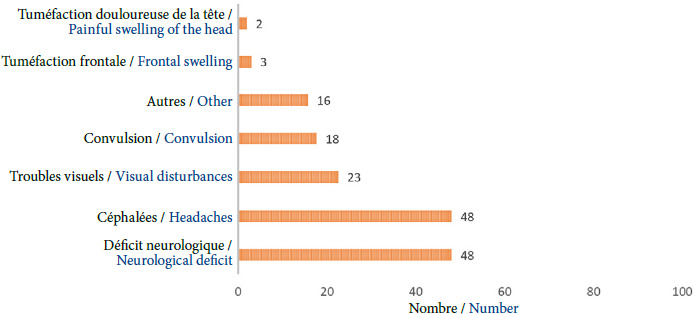
Description des symptômes révélateurs chez les patients suspectés d’une tumeur cérébrale

Les hypothèses diagnostiques à l’issue des examens cliniques et de l’imagerie sont exposées dans le Tableau I.

**Tableau I:** Répartition des cas selon les examens d’imagerie réalisés et les diagnostics paracliniques retenus

		**Effectifs**	**%**
**Type d’examen neuroradiologique réalisé**	TDM	94	92,2
	IRM	7	6,9
	Radiographie et IRM	1	1
**Diagnostic neuroradiologique évoqué**	Méningiome	40	39,2
	Tumeur cérébrale	34	33,3
	Gliome	15	14,7
	Épendymome	5	4,9
	Craniopharyngiome	4	3,9
	Processus expansif intracrânien	2	2
	Astrocytome pilocytaire	1	1
	Oligodendrogliome	1	1
	**Total**	**102**	**100**

**Figure 3:**
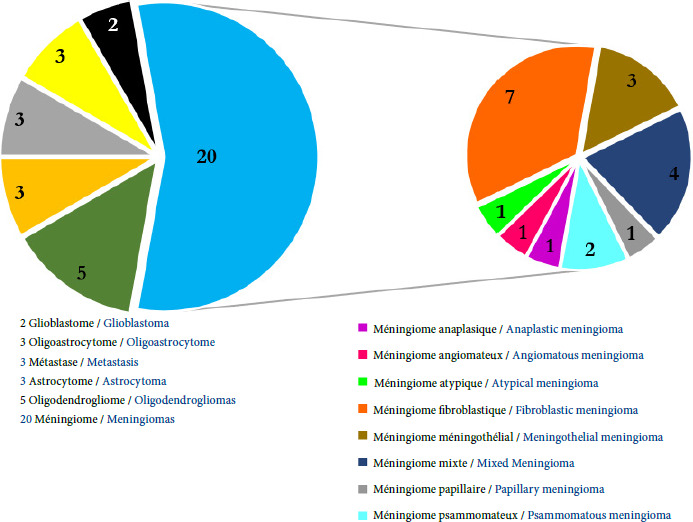
Répartition des patients selon les résultats histopathologiques

Trente-six patients (35,3%) ont réalisé un examen histopathologique. La majorité d’entre eux était des méningiomes (Fig. 3).

Le méningiome fibroblastique et le méningiome mixte étaient les méningiomes les plus retrouvés avec respectivement 35% et 20%, suivis des méningiomes méningothéliaux (15%), des méningiomes psammomateux (10%) et des méningiomes anaplasiques, angiomateux, atypiques et papillaires qui étaient retrouvés dans 5% des cas chacun.

La majorité des méningiomes était de grade 1 avec 17 cas (85%), suivi du grade 3 pour 2 cas (10%) et enfin du grade 2 (1 cas, soit 5%).

Sur les 20 cas de méningiomes diagnostiqués par l’histopathologie, l’imagerie médicale n’avait suspecté un méningiome que dans 5 cas (25%) et avait conclu à une tumeur cérébrale (60%), un gliome et un épendymome dans respectivement 10% et 5% des cas (Tableau II).

**Tableau II:** Description des diagnostics paracliniques retenus chez les patients avec méningiome

		**Effectifs**	**%**
**Diagnostic neuroradiologique évoqué**	Méningiome	5	25,0
	Tumeur cérébrale	12	60,0
	Gliome	2	10,0
	Épendymome	1	5,0
	**Total**	**102**	**100**

## Discussion

Nous avons mené cette étude dans l’objectif de déterminer l’apport de l’histopathologie dans le diagnostic des méningiomes intracrâniens dans les ESS de la ville de Kinshasa.

Le déficit neurologique, les céphalées, l’atteinte du nerf optique ainsi que les convulsions étaient liés aux méningiomes, à l’exception d’un patient ayant présenté un glioblastome pléomorphe associé à une atteinte du nerf optique. Il est important de noter que la fréquence et la nature des symptômes peuvent varier en fonction de plusieurs facteurs, tels que la localisation de la tumeur, sa taille, son grade histologique et la présence d’un œdème cérébral associé. Les méningiomes de grade élevé peuvent avoir une croissance plus rapide et être plus agressifs, ce qui peut entraîner des symptômes plus précoces et plus sévères. Cette symptomatologie a également été trouvée dans les études réalisées par Alvernia *et al.* et Oka *et al.,* suggérant que ces symptômes sont les manifestations cliniques les plus courantes des méningiomes intracrâniens [1,13]. Le déficit neurologique peut se manifester de différentes manières: troubles moteurs (parésie, paralysie), troubles sensitifs (paresthésies, hypoesthésie), troubles de l’équilibre et de la coordination. Les céphalées sont souvent liées à l’hypertension intracrânienne ou à la compression des structures nerveuses. L’atteinte du nerf optique, bien que moins fréquente dans notre série, est une complication potentielle des méningiomes situés à proximité de ce nerf. Elle peut entraîner une baisse de l’acuité visuelle. Les convulsions sont une autre manifestation possible des méningiomes, en particulier ceux qui se développent près du cortex cérébral et de la convexité sus-tentorielle [12,13,15,16].

Nos résultats sont globalement en accord avec les données de la littérature, qui rapportent que le méningiome est l’une des hypothèses diagnostiques les plus fréquemment évoquées en présence de signes cliniques et radiologiques suggestifs [10]. Cependant, la proportion exacte de chaque hypothèse peut varier en fonction de plusieurs facteurs, tels que la population étudiée, les critères de recrutement, les pratiques cliniques locales et l’accès aux techniques d’imagerie. D’autres hypothèses diagnostiques, telles que les tumeurs cérébrales, les processus expansifs intracrâniens et les tumeurs gliales, soulignent la nécessité d’un diagnostic différentiel rigoureux et multidisciplinaire. Les méningiomes peuvent parfois présenter des caractéristiques cliniques ou radiologiques qui les rendent difficiles à distinguer d’autres types de lésions intracrâniennes.

La collaboration entre cliniciens, radiologues et anatomopathologistes est essentielle pour établir un diagnostic précis afin de planifier une prise en charge adaptée. Toutefois, les radiologues doivent être vigilants sur les choix des techniques et examens neuroradiologiques dans le cadre du bilan des processus expansifs intracrâniens.

Notre étude met en évidence que l’examen histopathologique n’est pas systématiquement réalisé en cas de suspicion de tumeur cérébrale ou de méningiome. La divergence entre les résultats d’imagerie et ceux de l’histopathologie doit être analysée en profondeur afin d’améliorer la prise en charge de ces patients.

Les analyses histopathologiques devraient être réalisées systématiquement lorsqu’une intervention chirurgicale est effectuée afin de confirmer ou d’infirmer le diagnostic, permettant ainsi de déterminer le grade et un suivi approprié [3]. L’absence de biopsie chez les patients ayant subi une intervention chirurgicale, résulte généralement du coût de l’examen anatomopathologique: il n’est pas subventionné et est inaccessible à la plupart des patients. Il est donc urgent de mettre en place des politiques de santé publique efficaces dans ce domaine afin d’améliorer la prise en charge de ces patients, qui ne doit pas se limiter à l’intervention chirurgicale.

La connaissance du grade histologique des méningiomes est essentielle pour la prise en charge des patients. Les méningiomes de grade élevé nécessitent une prise en charge plus agressive, qui peut inclure une résection chirurgicale complète, une radiothérapie et/ou une chimiothérapie [7]. Les méningiomes de grade 1 sont généralement considérés comme des tumeurs bénignes, à croissance lente, avec un faible risque de récidive après résection chirurgicale complète [9]. Cependant, la présence de méningiomes de grade 3 dans notre étude souligne l’importance de ne pas négliger la possibilité de tumeurs plus agressives, à croissance rapide et à risque élevé de récidive et de métastases [2].

Nous suggérons de mettre en place des réunions de concertation pluridisciplinaire de neurooncologie pour discuter des dossiers de patients porteurs de tumeurs cérébrales, d’alimenter un registre des tumeurs cérébrales (épidémiologie) et d’assurer un suivi personnalisé pour chaque patient sur la base des résultats des examens neuroradiologiques et des données histopathologiques.

Les limites principales de notre étude résultent de la taille modeste de notre échantillon et du type rétrospectif de l’étude réalisée. Les données n’ont pas été collectées de façon standardisée et sont, pour certaines variables, lacunaires en raison des dossiers incomplets.

Il est important dans l’avenir de réaliser des études analytiques afin d’identifier les facteurs associés aux écarts des résultats entre l’imagerie et l’histopathologie dans notre milieu d’étude. Nous espérons avoir sensibilisé les différentes parties prenantes sur le bien-fondé d’une approche multidisciplinaire dans la prise en charge des tumeurs cérébrales en général et des méningiomes en particulier.

## Conclusion

L’examen histopathologie reste l’examen de référence dans le diagnostic des méningiomes. La détermination du grade histologique des méningiomes est essentielle pour une meilleure prise en charge et une surveillance appropriée. Cependant, malgré son importance, nous avons observé que la majorité de nos patients n’y avait pas recours en raison de son coût qui le rend inaccessible. L’écart important entre les conclusions de l’imagerie et celles de l’histopathologie concernant les méningiomes nécessite une sensibilisation des différents spécialistes. L’analyse approfondie de la situation permettra une prise en charge multidisciplinaire bénéfique aux patients. Cela pourrait être le résultat de réunions de concertation pluridisciplinaire de neuro-oncologie.

## Remerciements

Nous tenons à exprimer notre profonde gratitude envers les autorités académiques des cliniques universitaires de Kinshasa, tous les personnels et institutions qui ont contribué à la réalisation de cette étude. Parmi eux, ceux du département d’anatomie-pathologique de la faculté de médecine de l’université de Kinshasa, des CUK et de la clinique Ngaliema en RDC. Remerciements particuliers aux Dr Fraste Kaswij Muswiya et Ir Martin Mutuza Bakuzeza.

## Clairance éthique

Avant la collecte des données, notre recherche avait reçu l’approbation du comité éthique de l’université de Kinshasa et dans chaque ESS une autorisation des médecins directeurs était requise. Nous avons assuré la confidentialité des personnes ayant fait partie de notre étude.

## Financement

La réalisation de cette étude n’a reçu aucun financement quelconque.

## Contributions des auteurs

Conceptualisation: E. NKAMBA, O. J-M. KABONGO, G. NTSAMBI, B. LEB-WAZE Conservation des données: E. NKAMBA

Analyse des données: E. NKAMBA, O. KISILE, G. NTSAMBI

Méthodologie: E. NKAMBA, O. KISILE, G. NTSAMBI, R CHIRIMWAMI

Supervision de l’étude: O. KISILE, G. NTSAMBI, J-M. KABONGO, B. LEBWAZE, R CHIRIMWAMI

Rédaction: E. NKAMBA, O. KISILE, R CHIRIMWAMI, G. NTSAMBI, J-M. KABONGO

Approbation de la version finale: E. NKAMBA, O. KISILE, J-M. KABONGO, G. NTSAMBI, B. LEBWAZE, R. CHIRIMWAMI

## Déclaration de liens d’intérêt

Les auteurs ont déclaré n’avoir aucun lien d’intérêts associé à cette étude.
