# Supplementary material for: The role of histopathology in the diagnosis and management of intracranial meningiomas. Hospital experience in Kinshasa (Democratic Republic of Congo)
Source: Med Trop Sante Int. 2025 Oct 22;5(4):mtsi.v5i4.2025.647. [Article in French] doi: 10.48327/mtsi.v5i4.2025.647 (PMC12957992; doi:10.48327/mtsi.v5i4.2025.647)
Supplement: Related texts

# Research article: The role of histopathology in the diagnosis and management of intracranial meningiomas. Hospital experience in Kinshasa (Democratic Republic of Congo)

## Introduction

Meningiomas originate from the meningothelial cells of the arachnoid membrane and are the most prevalent primary non-glial brain tumors. They are considered the most common benign intracranial tumors [6]. The overall incidence rate has increased in USA from 4.52 to 8.3 per 100,000 people between 1998-2002 and 20102014 [11]. This increase can be attributed to the introduction of new diagnostic techniques. The predominant increase has been among people over the age of 70, among whom the incidence is 3.5 times higher. Literature reports a higher incidence of meningiomas in women, ranging from 2 to 7 per 100,000 people, compared to 1 to 5 per 100,000 people in men [16].

In recent years, several treatments for meningiomas have been developed, including gene therapy, molecular chemotherapy, hormone therapy, and multi-beam radiation therapy. However, surgery remains the primary treatment for meningiomas in developing countries, where it heavily relies on computed tomography (CT) and magnetic resonance imaging (MRI). Hence, it is crucial to have imaging results that closely resemble reality [4,8]. MRI plays a crucial role in detecting lesions, assessing tumor-related complications, and establishing differential diagnoses for intracranial meningiomas. Additionally, imaging a meningioma allows for an accurate and reliable diagnosis in most cases [14]. Several studies have demonstrated an association between MRI characteristics and meningioma grade. These characteristics include the presence of necrosis or tumor hemorrhage, tumor-brain interface, heterogeneous tumor enhancement, and peritumoral cerebral edema [14]. The prognosis for meningioma depends on its location, histological grade at the time of diagnosis, extent of resection, and biological aggressiveness [5]. Histopathology remains the gold standard for the diagnosis and classification of meningiomas. It plays an important role in management and follow-up, allowing the grade and histological subtype to be determined [9].

Practitioners are confronted with cases presenting discrepancies between clinical data, imaging, and histopathology. For example, a high-grade meningioma may be discovered incidentally by MRI in an asymptomatic patient or one presenting with atypical clinical signs and unusual symptoms such as cognitive impairment, whereas imaging suggests a low-grade meningioma. This discrepancy between imaging and histology is possible, particularly in certain histological subtypes such as secretory meningioma, which can have misleading radiological features. Artifacts are possible in histological techniques, particularly during surgical excision or slide preparation, which can make histological interpretation difficult. However, there may also be discrepancies related to intra-tumor heterogeneity. Finally, a discrepancy between clinical presentation and histology is possible depending on variables such as the patient’s age, medical history, or the presence of comorbidities, which may influence the clinical presentation and progression of the tumor.

We conducted this study to determine the contribution of histopathology to the diagnosis and classification of intracranial meningiomas in referral hospitals in the city of Kinshasa, Democratic Republic of Congo (DRC) in comparison with different clinical and medical imaging diagnostic approaches.

## Materials and methods

From January 2012 to December 2022, we conducted a descriptive cross-sectional study in tertiary healthcare facilities (THFs), which offer specialized care and training for specialist physicians. We selected THFs with pathology and neurosurgery departments in Kinshasa (DRC). Our sample consisted of patients admitted to the neurosurgery department with a brain tumor who had undergone medical imaging (CT, MRI, and radiology). A total of 102 patient records were selected. Patients were included if their file was available in the department and if the file or the department’s patient registry contained all the relevant data. Patients who had undergone surgery were included.

The data from the patient files were entered into an electronic form (KoboCollect) comprising four sections:

- Sociodemographic characteristics (age and sex);
- Clinical characteristics (symptoms and diagnostic hypotheses);
- Paraclinical characteristics (imaging tests performed and results);
- Histopathological characteristics (histopathological results and histological subtypes).

The iconographic diagnostic criteria concerned medical imaging. Patients underwent imaging examinations, including CT scans to detect intracranial masses, MRIs to provide detailed images of soft tissue and differentiate meningiomas from other tumors, and X-rays to assess tumor bone extension.

For the pathology diagnostic criteria, tumor tissue samples were collected by surgical excision. These samples were then subjected to pathology analysis in a laboratory to confirm the meningioma diagnosis, determine the histological type, establish the meningioma grade, or make another diagnosis. Due to the absence of this data in the majority of records, we were unable to list the types of surgery performed according to the Simpson classification.

A histopathological analysis was performed using hematoxylin and eosin staining.

The data were analyzed using SPSS® 27 software after being extracted to Excel® 2016.

Qualitative variables were expressed as frequencies and proportions, and quantitative variables were expressed as means and standard deviations.

## Results

Of the 102 patients in the series, the mean age was 47 years (range 14-79), with the highest proportion in the 41-60 age group (46 patients, or 45.1%), followed by the over 61 age group (35%). Males were overrepresented, accounting for 60 of the patients (58.8%) (Fig. 1).

**Figure 1:** Distribution of meningioma patients by age group and gender

**Figure 2:** Description of revealing symptoms in patients suspected of having a brain tumor

The diagnostic hypotheses following clinical examinations and imaging are shown in Table I. Thirty-six patients (35.3%) underwent histopathological examination. The majority of these were meningiomas (Fig. 3).

**Figure 3:** Distribution of patients according to histopathological results

**Tableau I:** Table 1: Distribution of cases according to imaging tests performed and paraclinical diagnoses made

		**Number**	**%**
**Type of neuroradiological examination performed**	CT	94	92,2
	MRI	7	6,9
	X-ray and MRI	1	1
**Suggested neuroradiological diagnosis**	Meningioma	40	39,2
	Brain tumor	34	33,3
	Glioma	15	14,7
	Ependymoma	5	4,9
	Craniopharyngioma	4	3,9
	Intracranial expansive process	2	2
	Pilocytic astrocytoma	1	1
	Oligodendroglioma	1	1
	**Total**	**102**	**100**

The most common types of meningioma were fibroblastic and mixed, accounting for 35% and 20% of cases, respectively. The next most common types were meningothelial (15%), psammomatous (10%), and anaplastic, angiomatous, atypical, and papillary (5% each).

Most meningiomas were grade 1 (17 cases, or 85%), followed by grade 3 (2 cases, or 10%), and grade 2 (1 case, or 5%).

Of the 20 meningioma cases diagnosed by histopathology, medical imaging suspected a meningioma in only five cases (25%) and concluded a brain tumor in six cases (30%), a glioma in two cases (10%), and an ependymoma in one case (5%) (Table II).

**Tableau II:** Table II: Description of paraclinical diagnostics used in patients with meningioma

		**Number**	**%**
**Suggested neuroradiological diagnosis**	Meningioma	5	25,0
	Brain tumor	12	60,0
	Glioma	2	10,0
	Ependymoma	1	5,0
	**Total**	**102**	**100**

## Discussion

The aim of this study was to determine the contribution of histopathology to the diagnosis of intracranial meningiomas in THFs in Kinshasa. Meningiomas were associated with neurological deficits, headaches, optic nerve damage, and seizures, except for one patient who presented with pleomorphic glioblastoma and optic nerve damage. It is important to note that the frequency and nature of symptoms may vary depending on several factors, including the tumor’s location and size, its histological grade, and the presence of associated cerebral edema. High-grade meningiomas tend to grow faster and are more aggressive, leading to earlier and more severe symptoms. Studies by Alvernia *et al.* and Oka *et al.* also found these symptoms, suggesting they are the most common clinical manifestations of intracranial meningiomas [1,13]. Neurological deficits can manifest in various ways, including motor disorders (paresis or paralysis), sensory disorders (paresthesia or hypoesthesia), or balance and coordination disorders. Headaches are often related to intracranial hypertension or compression of nerve structures. Although less common in our series, optic nerve involvement is a potential complication of meningiomas located near this nerve. It can lead to decreased visual acuity. Seizures are another possible manifestation of meningiomas, particularly those that develop near the cerebral cortex and supratentorial convexity [12,13,15,16].

Our results are consistent with previous studies that report meningioma as one of the most frequently considered diagnoses in cases with suggestive clinical and radiological signs [10]. However, the exact proportion of each hypothesis can vary based on factors such as the population studied, recruitment criteria, local clinical practices, and access to imaging techniques. Other diagnostic hypotheses, such as brain tumors, intracranial expansive processes, and glial tumors, underscore the necessity of rigorous, multidisciplinary differential diagnosis. Meningiomas can present features that are difficult to distinguish from other types of intracranial lesions, either clinically or radiologically.

Collaboration between clinicians, radiologists, and pathologists is essential for an accurate diagnosis and appropriate management planning. Nevertheless, radiologists must vigilantly select neuroradiological techniques and examinations when evaluating intracranial expansive processes. Our study shows that a histopathological examination is not routinely performed when a brain tumor or meningioma is suspected. Discrepancies between imaging and histopathology results must be analyzed to improve management of these patients.

Histopathological analyses should be systematically performed when surgery is performed to confirm or rule out the diagnosis and determine the grade, thereby enabling appropriate follow-up [3]. The absence of biopsies in patients who have undergone surgery is generally due to the cost of pathological examinations, which are not subsidized and are inaccessible to most patients. Therefore, there is an urgent need to implement effective public health policies to improve the management of these patients, which should extend beyond surgery.

Knowing the histological grade of a meningioma is essential for managing the patient. High-grade meningiomas require more aggressive treatment, which may include complete surgical resection, radiation therapy, and/or chemotherapy [7]. Grade 1 meningiomas are generally considered benign and slow-growing with a low recurrence risk after complete resection [9]. However, grade 3 meningiomas in our study underscore the importance of considering the possibility of aggressive, fast-growing tumors with a high recurrence and metastasis risk [2].

We recommend establishing multidisciplinary neuro-oncology consultation meetings to discuss cases of patients with brain tumors. These meetings should maintain a brain tumor registry and ensure personalized follow-up for each patient based on neuroradiological examination and histopathological data results.

The main limitations of our study stem from the small sample size and retrospective design. The data were not collected in a standardized manner, and some variables are incomplete due to incomplete records.

In the future, it is important to conduct analytical studies to identify factors associated with discrepancies between imaging and histopathology results in our study setting. We hope to raise awareness among various stakeholders of the benefits of a multidisciplinary approach to managing brain tumors, particularly meningiomas.

## Conclusion

A histopathological examination is still considered the gold standard for diagnosing meningiomas. Determining the histological grade of meningiomas is essential for better management and monitoring. However, despite its importance, we found that most of our patients did not undergo this examination due to its cost. The significant discrepancy between imaging and histopathology conclusions regarding meningiomas requires awareness among various specialists. An in-depth analysis of the situation will enable multidisciplinary management that benefits patients. This could result from multidisciplinary neuro-oncology consultation meetings.

## Acknowledgments

We would like to express our deep gratitude to the academic authorities of the university clinics in Kinshasa and to all the staff and institutions that contributed to this study. These include the Department of Anatomical Pathology at the University of Kinshasa’s Faculty of Medicine, the CUK, and the Ngaliema Clinic in the DRC. Special thanks to Drs. Fraste Kaswij Muswiya and Ir Martin Mutuza Bakuzeza.

## Ethical clearance

Before data collection, our research was approved by the University of Kinshasa’s ethics committee, and authorization from the medical directors was required at each THF. We ensured the confidentiality of the individuals who participated in our study.

## Funding

This study did not receive any funding.

## Authors’ contributions

Conceptualization: E. NKAMBA, O. J-M. KABONGO, G. NTSAMBI, B. LEB-WAZE Data Preservation: E. NKAMBA

Data analysis: E. NKAMBA, O. KISILE, G. NTSAMBI

Methodology: E. NKAMBA, O. KISILE, G.

NTSAMBI, R. CHIRIMWAMI

Study supervision: O. KISILE, G. NTSAMBI, J-M.

KABONGO, B. LEBWAZE, R. CHIRIMWAMI

Writing: E. Nkamba, O. Kisile, R. Chirimwami,

G. Ntsambi, J.-M. KABONGO

Approval of final version: E. NKAMBA, O. KISILE,

J-M. KABONGO, G. NTSAMBI, B. LEBWAZE, R.

CHIRIMWAMI

## Declaration of interests

The authors declared that they had no conflicts of interest associated with this study.
